# Disease trajectories and lived experiences of patients with X-linked hypophosphatemia in Taiwan: a qualitative study

**DOI:** 10.1093/jbmrpl/ziag163

**Published:** 2026-10-13

**Authors:** Shih-Yao Liu, Yi-Ching Tung, Chia-Chi Hsu, Yu-Yuan Ke, Ying-Hua Huang, Tun-Ying Hsu, Annie Chin, Ben Johnson, Wen-Yu Tsai

**Affiliations:** Department of Pediatric Endocrinology, National Taiwan University Hospital, 100 Taipei, Taiwan; Department of Pediatric Endocrinology, National Taiwan University Hospital, 100 Taipei, Taiwan; Department of Pediatric Genetics and Metabolism, Taichung Veterans General Hospital, 407219 Taichung, Taiwan; Department of Pediatric Genetics and Metabolism, Taichung Veterans General Hospital, 407219 Taichung, Taiwan; Department of Pediatric Genetics and Endocrinology, Kaohsiung Chang Gung Memorial Hospital, 833401 Kaohsiung, Taiwan; Healthcare, DKSH Management Pte. Ltd. (Asia Pacific), 329983 Singapore Singapore; Medical Affairs, Kyowa Kirin Taiwan Co., Ltd., 10448 Taipei City, Taiwan; Department of Health Economics and Outcomes Research, Kyowa Kirin International plc, SL7 1HZ Marlow, United Kingdom; Department of Pediatric Endocrinology, National Taiwan University Hospital, 100 Taipei, Taiwan

**Keywords:** X-linked hypophosphatemia, qualitative research, phosphate, patient-reported outcomes, rare disease, Taiwan

## Abstract

X-linked hypophosphatemia (XLH) is a rare, lifelong disorder of renal phosphate wasting that typically presents in childhood with skeletal deformities, growth retardation, and functional impairment, although these features may be less pronounced in milder cases. The disease persists into adulthood with chronic pain, mobility limitations, and psychosocial challenges. While pediatric XLH has been well described internationally, the lifelong disease journey in Taiwan remains poorly characterized. We conducted a multicenter qualitative study using semi-structured, in-depth interviews between July 2024 and January 2025 at 3 major medical centers, enrolling 15 patients with a clinical diagnosis of XLH (genetically confirmed in a subset): 8 pediatric patients (aged ≥5 yr) interviewed with caregivers and 7 adults interviewed independently. Interviews (~60 min) explored diagnostic pathways, treatment experiences, disease burden, and support needs; transcripts were analyzed thematically. Participants commonly reported delayed or missed diagnosis, with early symptoms such as bowed legs and short stature often misattributed to nutritional or growth-related issues, leading to late referral to specialist care. Oral phosphate supplementation and active vitamin D (Pi/D) were the predominant treatments; however, frequent dosing, poor palatability, and gastrointestinal side effects contributed to low adherence. Many adults had discontinued therapy, and 12 patients underwent corrective limb surgery, mainly during childhood or adolescence. Across the life course, patients experienced chronic bone pain, reduced mobility, and insufficiency fractures, as well as psychosocial burdens, including anxiety, low self-esteem, social withdrawal, and caregiver stress, often compounded by guilt related to hereditary transmission. Patients and caregivers emphasized unmet needs in disease education, transition from pediatric to adult care, peer support, and interest in novel therapies, such as burosumab. These findings highlight substantial lifelong disease burden among patients with XLH in Taiwan and underscore the need for earlier diagnosis, multidisciplinary care, and improved access to emerging therapies.

## Introduction

X-linked hypophosphatemia (XLH) is a rare, inherited disorder of renal phosphate wasting caused by pathogenic variants in the *PHEX* gene, leading to elevated fibroblast growth factor 23 (FGF23) and impaired phosphate homeostasis.^1^ X-linked hypophosphatemia typically presents in childhood with bowing of the legs, short stature, delayed walking, and rickets, and persists into adulthood with osteomalacia, chronic bone and joint pain, enthesopathy, dental abnormalities, and impaired physical function.^2^^,^^3^ Beyond musculoskeletal manifestations, patients often experience psychosocial challenges, including reduced self-esteem, anxiety, and limitations in educational attainment, employment, and social participation, underscoring its lifelong burden.^4^^,^^5^

While the clinical phenotype of pediatric XLH has been well described, there is limited research on patients’ lived experiences across the life course. Although several qualitative studies conducted in North America and Europe have described the lived experiences, psychosocial burden, and treatment challenges faced by patients with XLH, most have focused on pediatric populations or cross-sectional perspectives.^6^^,^^7^ The majority of previous studies have also emphasized biochemical and radiographic outcomes and the clinical efficacy of burosumab.^8^^,^^9^

Consequently, data capturing the longitudinal, life-course experience of patients—particularly among adult populations and in Asian settings—remain limited. This gap is notable given growing recognition that XLH requires holistic, multidisciplinary care throughout life.^4^^,^^10^ The 2022 international XLH consensus statement further highlighted the importance of improving transition from pediatric to adult care, addressing psychosocial needs, and ensuring equitable access to emerging therapies.^11^

Given Taiwan’s unique context—underpinned by the Rare Disease and Orphan Drug Act, national insurance coverage, rare disease subsidy programs, and a regional rare disease care network—healthcare pathways and lived disease journeys for XLH patients may differ from those described in Western cohorts, particularly in terms of referral patterns, access to specialized care, and levels of disease awareness among non-specialist physicians. The Act has significantly increased orphan drug availability and patient access through the National Health Insurance system.^12^ Moreover, while Asia–Pacific XLH consensus recommendations emphasize the importance of transitional care, most supporting data are derived from studies conducted outside the Asia–Pacific region,^13^ highlighting the lack of Taiwan-specific insights.

The objective of this study was therefore to explore the lifelong experiences of patients with XLH in Taiwan, from childhood through adulthood, including disease burden, treatment experiences, and psychosocial impacts, using a multicenter, thematic qualitative approach. By documenting patients’ voices, this study aims to identify unmet needs, support the development of patient-centered care, and provide evidence to guide future clinical practice and health policy in Taiwan.

## Materials and methods

### Study design and setting

This qualitative, multicenter interview study was conducted at three major medical centers in Taiwan: National Taiwan University Hospital (NTUH, Taipei), Taichung Veterans General Hospital (TVGH, Taichung), and Kaohsiung Chang Gung Memorial Hospital (KCGMH, Kaohsiung). Principal investigators at each site were specialists in pediatric endocrinology and genetics, ensuring clinical expertise in patient identification and study oversight.

### Participants and recruitment

A total of 15 patients with XLH (8 pediatric and 7 adult) were enrolled between July 2024 and January 2025. Eligible patients were identified and invited by treating physicians at participating centers based on clinical records and ongoing care in specialized endocrinology and genetics clinics. Pediatric patients were accompanied by caregivers who provided additional input. Caregivers were interviewed to provide supplementary perspectives but were not considered independent study participants; the study sample size reflects only individuals diagnosed with XLH. Written informed consent was obtained from all adult participants and from caregivers of pediatric patients, with assent from children as appropriate.

Inclusion criteria included a diagnosis of XLH based on clinical, biochemical, and/or genetic evidence, age ≥5 yr for pediatric participants, and no current participation in any randomized controlled trial. Genetic confirmation of pathogenic PHEX variants was available for a subset of participants (*n* = 8); details of the identified variants are provided in Table S1. For the remaining participants, diagnosis was established by experienced endocrinology or genetics specialists based on characteristic clinical features (eg, lower limb deformities and short stature), biochemical findings consistent with renal phosphate wasting, and/or family history, reflecting routine clinical practice in real-world settings. Exclusion criteria included prior or current use of burosumab and inability or refusal to provide informed consent.

### Data collection

In-depth, semi-structured interviews were conducted with each participant. Pediatric patients were interviewed together with their caregivers, while adult patients were interviewed independently. Each interview lasted approximately 60 min, was audio-recorded with permission, and transcribed verbatim. The interview guide explored key domains of the XLH disease journey, including clinical experiences, treatment challenges, psychosocial impact, and unmet needs (Table S2).

### Data analysis

Transcripts were analyzed using thematic analysis. Two independent coders reviewed transcripts and developed an initial coding framework based on both deductive themes (derived from the interview guide) and inductive themes (emerging from the data). Coding discrepancies were resolved by consensus. Themes were refined iteratively to capture recurring patterns across pediatric and adult patients. Participant recruitment continued until thematic saturation was achieved, defined as the point at which no new themes emerged across the final interviews.

### Ethics

This study was approved by the Institutional Review Boards of NTUH (approval numbers: NTUH-REC No. 202402041RSD, Date of approval: April 26, 2024), TCVGH (approval numbers: SF24097B, Date of approval: February 15, 2024), and KCGMH (approval numbers: 202400539A3, Date of approval: June 13, 2024). Written informed consent was obtained from all adult participants and from caregivers of pediatric participants, with assent from children as appropriate. Interviews were conducted in Mandarin. Quotations were translated into English and back-translated by bilingual researchers to preserve meaning; all transcripts were de-identified prior to analysis.

## Results

### Participant characteristics

A total of 15 patients with XLH were interviewed, including 8 pediatric patients (aged 8-18 yr) and 7 adults (aged 27-61 yr). Participants were recruited from 3 tertiary referral centers in Taiwan: National Taiwan University Hospital (*n* = 8), Taichung Veterans General Hospital (*n* = 6), and Kaohsiung Chang Gung Memorial Hospital (*n* = 1). The cohort was predominantly female (*n* = 11), with heights ranging from 124 to 155 cm. Educational backgrounds ranged from primary school to postgraduate level, reflecting heterogeneity in social participation and life opportunities. Among adults, several were single and without children. Pediatric patients were accompanied by caregivers (mostly parents) during the interviews. Anthropometric participant characteristics are summarized in Table 1. Height, weight, and body mass index standard deviation scores (SDS) were calculated using age- and sex-specific reference data derived from Taiwanese growth charts.^14^

**Table 1:** Participant characteristics of the study cohort.

	**Pediatric (*n* = 8)**	**Adult (*n* = 7)**	**Total (*N* = 15)**
**Age, years**	8-18	27-61	8-61
**Gender, *n* (%)**	Female 4 (50%), Male 4 (50%)	Female 7 (100%)	Female 11 (73%), Male 4 (27%)
**Height, cm**	124-149	142-155	124-155
**Height SDS** ^a^	−2.73 ± 1.64	−2.11 ± 1.04	−2.44 ± 1.38
**Weight, kg**	28-63	39-88	28-88
**Weight SDS**	−0.41 ± 1.08	0.45 ± 1.79	−0.01 ± 0.12
**BMI, kg/m** ^2^	18.2-26.2	18.5-40.2	18.2-40.2
**BMI SDS**	1.75 ± 2.47	1.26 ± 2.17	1.52 ± 1.67
**Education level**	Primary school (elementary) (*n* = 3), Junior high school (*n* = 3), and Senior High School (*n* = 2)	Bachelor’s degree (*n* = 4), Master’s degree (*n* = 2), and Technical School (*n* = 1)	Primary–Master
**Marital status**	NA (pediatric)	Single (*n* = 4), Married (*n* = 1), Divorced (*n* = 1), and Unmarried (*n* = 1)	–
**Number of children**	NA	Two participants had children	–

a SDS, standard deviation score; calculated using age- and sex-specific Taiwanese growth reference data.^14^

### Diagnosis journey

Most patients first presented with gait abnormalities or lower limb deformities during early childhood, most commonly between 1 and 6 yr of age. The mean interval between symptom onset and first medical consultation was 1.3 yr, followed by an additional 1.2 yr from the first consultation to confirmed diagnosis. Patients typically consulted 2-4 physicians before receiving a definitive diagnosis of XLH. Orthopedic surgeons were most frequently the first specialists consulted, whereas fewer patients initially visited endocrinology, nephrology, or pediatric genetics departments. Several participants reported initial misdiagnoses, such as achondroplasia, growth retardation, or isolated knee deformities, which contributed to delays in appropriate treatment initiation (Figure 1).

**Figure 1:**
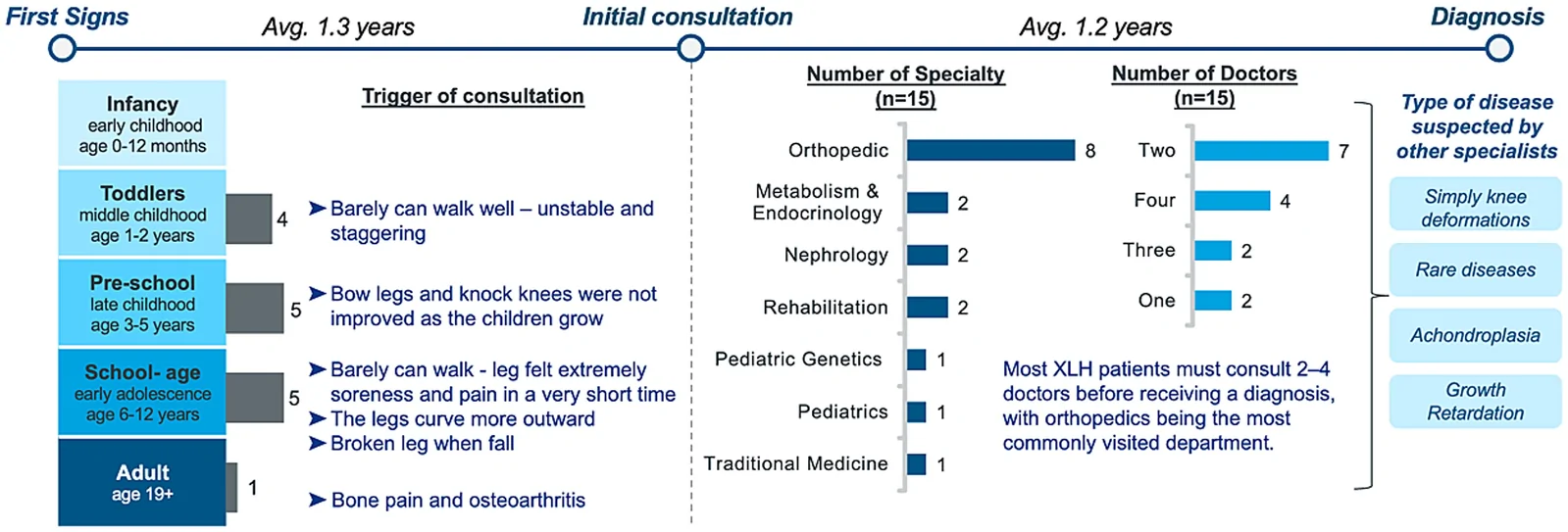
Diagnostic journey of XLH patients in Taiwan. Average time from first symptom onset to initial consultation was 1.3 yr, followed by an additional 1.2 yr from first consultation to confirmed diagnosis. Patients consulted a median of 2-4 doctors before diagnosis, with orthopedic surgeons being the most common initial specialty visited. Misdiagnoses (eg, achondroplasia, growth retardation, or isolated knee deformities) were frequently reported.

These delays were commonly attributed by participants to initial consultations with non-specialists, misinterpretation of early symptoms as normal growth variation, and limited awareness of XLH among healthcare providers, suggesting that delayed diagnosis may reflect changes in disease awareness and referral practices over time rather than generational differences. From a healthcare system perspective, in Taiwan, genetic services are not formally separated into pediatric and adult specialties; however, differences in physician training backgrounds may influence referral patterns and clinical pathways.

Among pediatric participants, the mean age at diagnosis was approximately 3.5 yr (range: 2-5 yr), typically following recognition of bowed legs or abnormal gait by caregivers. Parents often sought medical advice after noticing walking difficulties between 1 and 3 yr of age, and diagnosis was usually established after consultation with orthopedic specialists.

### Current management experiences

All participants reported receiving phosphate supplementation and active vitamin D (Pi/D)-based therapy, a regimen that has long been recognized to have limited efficacy and to be associated with adherence challenges and treatment-related complications.^15^^,^^16^ While Pi/D therapy aims to improve biochemical abnormalities—primarily by reducing alkaline phosphatase levels and maintaining PTH within the normal range—complete normalization of serum phosphate is not typically achieved. Phosphate supplementation and active vitamin D may be a viable and less costly treatment option for patients with milder disease. Careful clinical management and regular monitoring are needed to minimize treatment-related adverse effects of Pi/D, such as nephrocalcinosis and secondary hyperparathyroidism. Satisfaction with treatment effectiveness was generally low to moderate, as reflected in patient-reported Likert scores (Figure 2A). Difficulties maintaining adherence were common, with gastrointestinal side effects, poor palatability, and the need for frequent dosing often every 4-6 h, up to 6 times daily reported as major barriers. Participants described these regimens as inconvenient and intrusive to daily routines. Perceived burden extended beyond medication itself, encompassing challenges in balancing school or work schedules and the frequency of hospital visits (Figure 2B-D).

**Figure 2:**
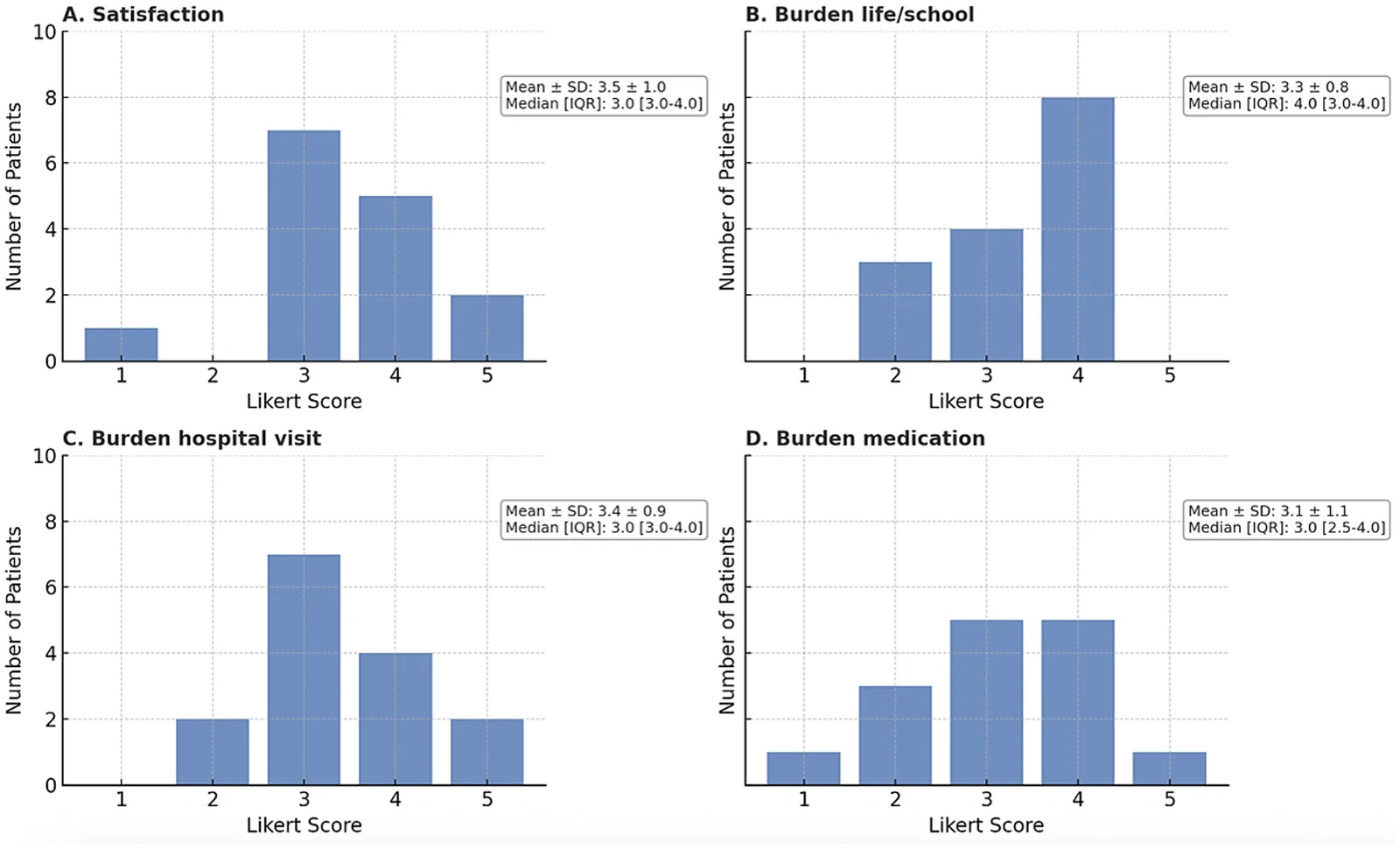
Patient-reported satisfaction and perceived burden related to treatment and daily life. (A) Overall satisfaction with current therapy in improving XLH symptoms, rated on a 5-point Likert scale (1 = very dissatisfied, 5 = very satisfied). (B-D) Perceived burden associated with (B) balancing school/work, (C) frequency of hospital visits, and (D) medication routines, each rated on a 5-point Likert scale (1 = very burdensome, 5 = not burdensome). Bars represent the number of patients reporting each score (*N* = 15).

Orthopedic interventions were common, with 12 patients (7 pediatric and 5 adult) undergoing corrective surgeries during adolescence, primarily for genu varum or genu valgum. Detailed information on surgical procedures, including type, age at intervention, and calendar year, is provided in Table S3. While the majority underwent 2 or 3 procedures, severe cases required as many as 10 surgeries, highlighting the significant physical burden of the disease. Recovery was described as prolonged and painful, often lasting 6-12 mo, and was perceived to significantly disrupt school attendance, work productivity, and social life. Dental care needs were also reported, with 6 patients requiring root canal treatment or dentures. Rehabilitation therapies (*n* = 4) and nephrology follow-up (*n* = 5) were sought mainly for mobility limitations and monitoring of treatment-related nephrocalcinosis. Among participants with available renal imaging data, nephrocalcinosis of at least mild severity was observed in the majority of cases (6/8), most commonly identified during childhood. No participants were reported to have CKD or to have undergone renal transplantation. Detailed individual-level findings, including age at detection and severity, are provided in Table S4.

### Disease burden across the lifespan

Patients described disease burden extending from childhood into adulthood. Pediatric patients frequently reported bowed legs, leg pain, and limitations in physical activities, leading to reduced participation in sports and peer interactions (Figure 3A). Adolescents highlighted issues of self-esteem and social isolation, especially when visible skeletal deformities elicited unwanted attention from classmates.

**Figure 3:**
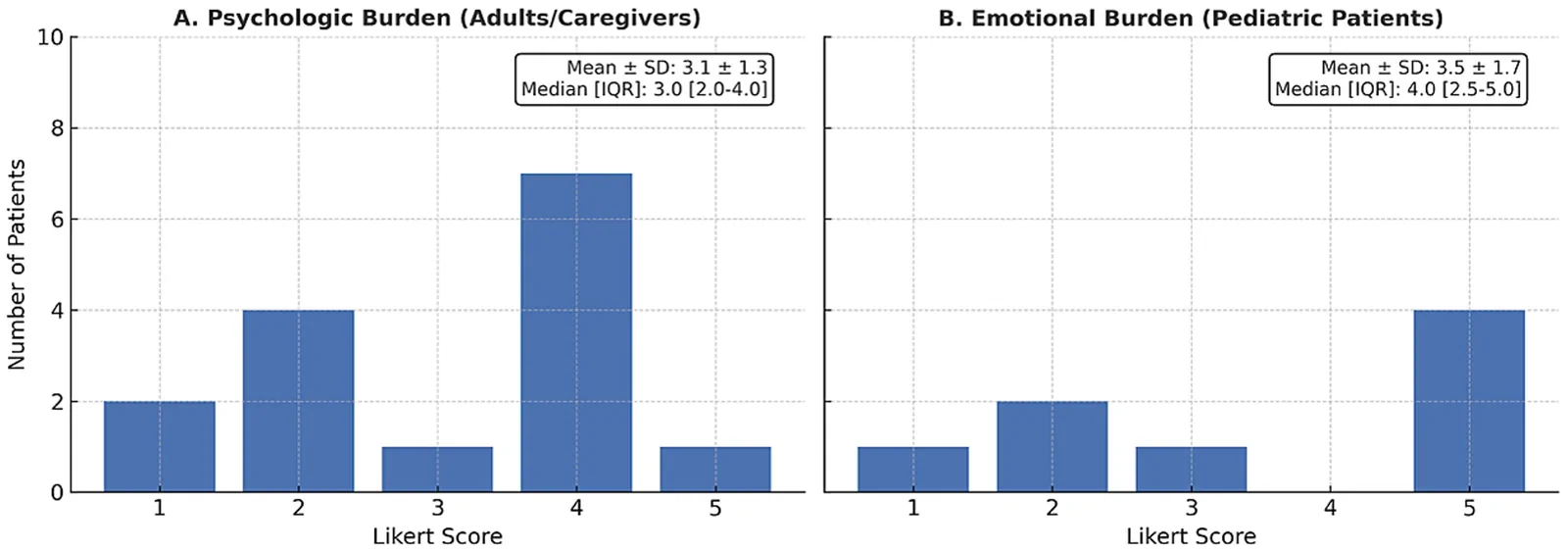
Psychological and emotional burden among patients with XLH and their caregivers. Patients and caregivers rated their recent psychological well-being using 2 Likert-scale questions: (A) Psychological burden living with XLH (*N* = 15 adult patients or caregivers), “How often have you been affected by negative emotions in recent days?” (1 = very often, 5 = never); and (B) emotional burden living with XLH—pediatric patients (*N* = 8), “To what extent has illness or physical discomfort influenced your mood recently?” (1 = very severe impact, 5 = no impact). Bars represent the number of respondents at each score. Mean ± SD and median [IQR] are shown in the upper right corner of each panel.

Adult patients reported persistent bone pain, fatigue, and reduced mobility despite long-term Pi/D therapy (Figure 3A). Several adults expressed concerns about dental deterioration, recurrent surgeries, and the risk of kidney calcification due to prolonged therapy. These symptoms adversely affected their ability to sustain employment and maintain daily function. Across the cohort, adherence to lifelong medication regimens was perceived as a constant reminder of illness, negatively impacting psychological well-being.

### Emotional and social impact

Interviewees consistently described emotional challenges, including anxiety, depression, and feelings of inferiority compared to peers (Figure 3B). Adolescents reported particular difficulties balancing treatment demands with schoolwork, while adults emphasized frustration with reduced work capacity and concerns about future independence. Caregivers expressed emotional strain related to long-term responsibility, anxiety about hereditary risk, and difficulty discussing the disease openly with their children (Figure 3B). Some caregivers also mentioned that certain family members perceived the condition as a stigma or a form of “bad karma” inherited from ancestors, reflecting deep-rooted cultural beliefs and guilt within the family.

### Medical education and patient voices

Patients and caregivers emphasized the need for more comprehensive and accessible educational resources, preferably written materials that explain disease mechanisms, treatment strategies, and lifestyle guidance. Participants noted that limited consultation times with healthcare providers often restricted opportunities to ask questions.

While many respondents supported the idea of establishing patient support groups (Figure 4A), enthusiasm for participation was mixed. Some welcomed the potential for shared experiences and peer encouragement, while others were reluctant due to fear of stigma, avoidance of disease-related reminders, or lack of perceived necessity (Figure 4B). Patients expressed a strong interest in new treatment options, such as burosumab, and highlighted the importance of integrated psychosocial support alongside medical management.

**Figure 4:**
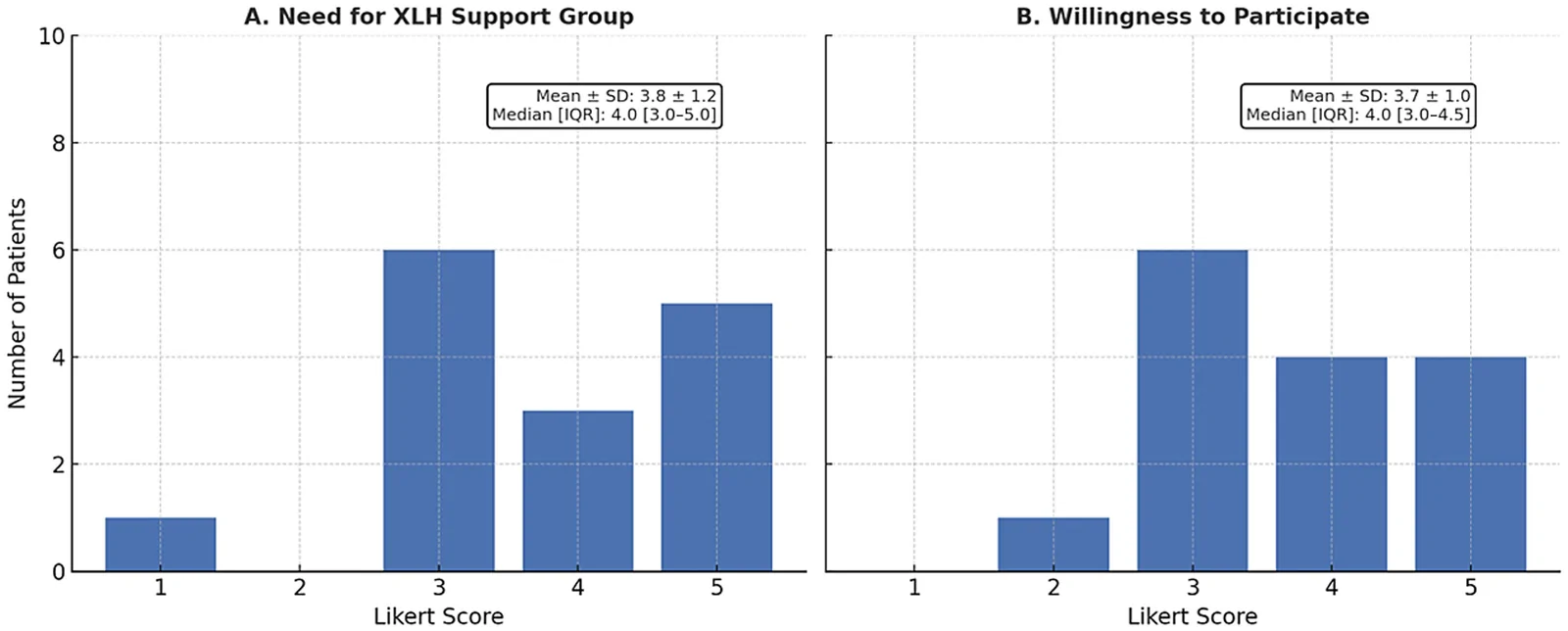
Patient and caregiver perspectives on XLH support groups. (A) Perceived need for establishing an XLH patient support group and (B) willingness to participate if such a group were available. Participants (*N* = 15) rated their responses on a 5-point Likert scale. For the first question, patients were asked: “Do you think there is a need to establish a local XLH patient support group or organization?” (1 = extremely unnecessary, 2 = unnecessary, 3 = neutral, 4 = necessary, and 5 = extremely necessary). For the second question, patients were asked: “If such a group were available, how willing would you be to participate?” (1 = extremely unwilling, 2 = unwilling, 3 = neutral, 4 = willing, and 5 = extremely willing). Reported considerations affecting willingness to participate included concerns about social exposure, perceived usefulness of peer exchange, and personal disease burden. Data are presented as frequency distributions, with mean ± SD and median [IQR] indicated within each panel.

## Discussion

This study highlights that XLH is not solely a pediatric disease but rather a lifelong condition that continues to affect patients well into adulthood. While bowed legs, impaired growth, and reduced mobility are prominent during childhood, many adult participants described persistent bone pain, dental deterioration, and reduced mobility that limited their ability to work and maintain daily function. These findings reinforce prior qualitative research documenting the progressive and multisystem burden of XLH across the lifespan,^6^^,^^10^ while extending the evidence by providing the first systematic qualitative account from Taiwan, contributing to the growing body of data from Asia–Pacific populations.

These findings also suggest that delayed diagnosis is not solely attributable to disease rarity but may reflect limited awareness of XLH in early-contact specialties, such as orthopedics, as well as misattribution of early symptoms to normal growth variation. Notably, diagnostic delay was observed even among individuals with a known family history, indicating gaps in disease recognition and referral pathways. Taken together, these findings highlight potential gaps in long-term follow-up and continuity of care, suggesting the importance of structured care strategies that bridge pediatric and adult services within regional healthcare systems.

While Pi/D therapy aims to improve biochemical markers—primarily by reducing alkaline phosphatase levels and maintaining PTH within the normal range—complete normalization of serum phosphate is not typically achieved. Despite widespread use of Pi/D, participants reported challenges related to treatment adherence. Participants frequently reported gastrointestinal discomfort, poor palatability, and the burden of frequent dosing schedules. Moreover, several adult participants reported dose interruption or reduced adherence related to frequent dosing demands, tolerability challenges, and perceived limited treatment benefit—a pattern consistent with long-standing clinical observations that Pi/D therapy may be constrained by dosing burden, tolerability issues, and treatment-related complications.^15^^,^^16^ In addition to medication-related challenges, recovery from orthopedic surgeries was described as painful and disruptive, echoing reports of high disease burden and impaired quality of life in adults with XLH.^17^ Dental care needs were also reported, with 6 patients requiring root canal treatment or dentures. These findings parallel documented dental manifestations in XLH patients—including spontaneous dental abscesses and structural defects in enamel and dentin—as shown in case descriptions and longitudinal evaluations.^18^ Furthermore, nephrocalcinosis related to prolonged phosphate and vitamin D supplementation remains a recognized complication of Pi/D therapy.^16^^,^^19^ Phosphate supplementation and active vitamin D therapy remains an effective treatment option, particularly for patients with milder disease manifestations, when appropriately monitored. Compared with burosumab, Pi/D therapy may also impose a lower economic burden, though careful clinical management and regular monitoring would be needed to mitigate treatment-related adverse effects.^20^

The burden of XLH extends well beyond its physical manifestations. Adolescents often experienced social isolation and low self-esteem due to visible skeletal deformities and treatment demands that interfered with school life. Adult patients expressed frustration over reduced work capacity, concerns about maintaining independence, and anxiety about passing the condition on to future generations. Caregivers likewise reported emotional stress associated with chronic responsibilities and difficulties in discussing the disease openly with their children. Similar psychosocial challenges—including low self-esteem, social withdrawal, and caregiver burden—have been reported in multi-country qualitative studies, underscoring the broader relevance of these themes.^6^^,^^10^

Cultural perceptions, such as beliefs linking XLH to ancestral karma or moral causality, may further exacerbate stigma and guilt within families. Such findings highlight the importance of culturally sensitive psychological counseling and community education in Asian contexts, where inherited diseases are sometimes perceived through moral or spiritual lenses.

Looking forward, patients and caregivers identified clear priorities for improving care. Many expressed strong interest in newer therapeutic options, including burosumab. Randomized controlled trials and long-term extension studies have demonstrated improvements in biochemical parameters, bone health, and patient-reported outcomes compared with Pi/D therapy. Real-world analyses further suggest potential benefits in mobility and quality of life, although issues of access, long-term safety, and reimbursement feasibility remain critical considerations.^21–25^

At the same time, participants emphasized the value of peer support networks and experience-sharing platforms as a means to reduce stigma, foster resilience, and enhance disease understanding. These priorities are aligned with international consensus recommendations that highlight the importance of transitional care, multidisciplinary collaboration, and patient education, though Taiwan-specific adaptations have not yet been established.^11^

Taken together, these findings may suggest potential gaps in long-term follow-up and continuity of care, supporting consideration of structured care strategies that bridge pediatric and adult services within regional healthcare systems.

Additionally, as participants were recruited from tertiary referral centers, the study population may overrepresent individuals with more severe disease, higher healthcare utilization, and greater treatment burden. This should be considered when interpreting the generalizability of the findings.

### Limitations

This study has several limitations. First, the sample size was small, reflecting the rarity of XLH, and findings may not be generalizable to all patients in Taiwan or other populations. Second, the qualitative design relied on self-reported experiences, which may be subject to recall bias or selective reporting. Third, although participants were recruited from three major referral centers, the perspectives of patients who are not regularly engaged in tertiary care may not have been captured. Finally, as the purpose of this study was to capture the natural disease course and patient experiences under Pi/D therapy, individuals receiving burosumab were intentionally excluded; therefore, the findings do not encompass experiences associated with newer treatment modalities.

### Conclusion

This qualitative study underscores that XLH exerts a lifelong impact that extends far beyond skeletal manifestations, affecting psychological well-being, social participation, and overall quality of life. Patient and caregiver narratives revealed substantial unmet needs related to treatment adherence, fragmented transition from pediatric to adult care, and limited psychosocial support. There is a critical need for health policies that facilitate early diagnosis, ensure continuity of care across the lifespan, and integrate multidisciplinary and patient-centered approaches. Expanding access to innovative therapies alongside holistic care models may represent a potential strategy to address these unmet needs. However, this should be interpreted as an author perspective informed by patient-reported experiences and existing literature, rather than a direct conclusion derived from the present qualitative dataset.

Studies related to the development of structured care pathways and referral systems for adult XLH management, the establishment of peer support networks to encourage experience sharing and psychosocial resilience, and the promotion of local clinical research to evaluate emerging therapies within the Taiwanese healthcare context can be considered in the future.

## Supplementary Material

Supplementary_Tables_ziag163

## Data Availability

The qualitative data generated and analyzed during this study are not publicly available due to the sensitive nature of the interview data and the need to protect participant confidentiality.
