# Supplementary material for: Disease trajectories and lived experiences of patients with X-linked hypophosphatemia in Taiwan: a qualitative study
Source: JBMR Plus. 2026 Oct 13;10(11):ziag163. doi: 10.1093/jbmrpl/ziag163 (PMC13695141; doi:10.1093/jbmrpl/ziag163)
Supplement: Supplementary file 1 [file supplementary_tables_ziag163.docx]

**Supplementary Table 1. Pathogenic variants in the PHEX gene among genetically confirmed participants**

| **Patient No.** | **Age** | **Sex** | **Exon** | **cDNA** | **Protein** |
| --- | --- | --- | --- | --- | --- |
| **01** | 18y 10m | M | 21 | c.2117_2135del | p.Gln706ProfsTer28 |
| **02** | 15y 11m | M | 16 | c.1650del | p.Pro551GlnfsTer19 |
| **03** | 36y 3m | F | 22 | c.2240G>A | p.Arg747Gln |
| **04** | 29y 6m | F | 1 | c.58C>T | p.Arg20Ter |
| **05** | 29y 4m | F | 22 | c.2148_2156delinsAA | p.Val717MetfsTer21 |
| **06** | 29y | F | 17 | c.1735G>A | p.Gly579Arg |
| **07** | 10y 2m | M | 1-2 | NC_000023.22: (?_22033001)-(22038561_?) del | Exon 1 to Exon 2 deletion |
| **08** | 11y 9m | F | 17 | c.1699C>T | p.Arg567Ter |

**Supplementary Table 2. Representative interview questions used in the qualitative study**

| Domain | Representative interview questions |
| --- | --- |
| 1. Basic information / Demographics | - Could you confirm your year of birth? - What is your current age? - What is your height and weight? - What is your highest level of education? - What is your biological sex? - What is your marital status? - Do you have children? If yes, how many and how old are they? |
| 2. Disease awareness and diagnosis | - How do you usually refer to this disease? - When were you diagnosed with this condition? - How old were you at the time of diagnosis? - How was the disease first identified? - What symptoms were noticed before diagnosis? - Who first noticed these symptoms (yourself or family members)? - Where was the diagnosis confirmed? - Did you experience any misdiagnosis or delayed diagnosis? |
| 3. Pre-diagnosis and care journey | - Before diagnosis, how well did you understand the symptoms? (1–5 scale) - Before diagnosis, how well did you understand the healthcare-seeking process? (1–5 scale) - Which healthcare providers did you visit before diagnosis? - What evaluations or treatments were performed before diagnosis? |
| 4. Treatment and follow-up | - How often do you attend follow-up visits? - What treatments have you received for this condition? - Have you ever used burosumab or phosphate-related therapy? - How well do you understand the treatment process? (1–5 scale) - How well do you understand your medication regimen? (1–5 scale) - Have you experienced any difficulties adhering to treatment? |
| 5. Daily life and caregiving | - How well do you understand or manage daily care related to this condition? (1–5 scale) - Do you require assistance from others for daily activities? - Has the disease affected your daily life or physical function? - Does it affect activities such as walking, running, or eating? |
| 6. Healthcare access and burden | - How long does it take to travel to the hospital? - What transportation do you usually use? - Do you find the travel time burdensome? - Are treatment-related costs covered by insurance? - Have you experienced financial burden due to this disease? |
| 7. Genetic testing and family history | - Have you undergone genetic testing? - What type of genetic testing was performed? - Do you recall the results? - Was a genetic mutation identified? - Are there other family members with this condition? - Is there any suspected hereditary pattern in your family? |
| 8. Comorbidities and symptoms | - Do you have any other medical conditions or comorbidities? - Have you experienced dental problems (e.g., caries, gingivitis)? - Are you currently experiencing pain? - Where is the pain located? - Under what conditions does the pain occur? - How severe or frequent is the pain? - How do you manage these symptoms? |
| 9. Healthcare utilization | - Have you consulted other specialists besides your primary physician? - Have you sought genetic counseling or rehabilitation services? - Have you visited other hospitals for this condition? |
| 10. Psychosocial impact | - In the past month, have you experienced stress or emotional distress related to this condition? - Has the disease affected your mood or daily activities? - Do you have concerns about family members or children with this condition? |

**Supplementary Table 3. Surgical history of participants with X-linked hypophosphatemia (XLH)**

| **Patient No.** | **Sex** | **Age at surgery** | **Year** | **Procedure** |
| --- | --- | --- | --- | --- |
| 01 | M | 8 | 2015 | Guided growth (medial distal femur and proximal tibia) |
|  |  | 10 | 2017 | Revision of guided growth (screw pull-out) |
|  |  | 12 | 2019 | Guided growth with 8-plate (revision/repositioning) |
|  |  | 13 | 2020 | Guided growth revision due to screw loosening (hinged plate) |
|  |  | 14 | 2021 | Guided growth revision due to screw loosening |
| 02 | M | 7 | 2017 | Lateral epiphysiodesis (distal femur and proximal tibia, 8-plate) |
|  |  | 11 | 2021 | Implant removal and varus guided growth (medial distal femur and proximal tibia) |
|  |  | 13 | 2023 | Guided growth with implant removal after correction |
| 04 | F | 15 | 2012 | Tibial and femoral osteotomy with internal fixation; fibular osteotomy |
|  |  | 16 | 2013 | Corrective osteotomy and ORIF (femur and tibia) |
|  |  | 18 | 2015 | Bilateral corrective osteotomy; residual varus correction |
| 05 | F | 12 | 2009 | Tibial and femoral lengthening with external fixation (Ilizarov/Orthofix) |
|  |  | 13 | 2009 | Corrective osteotomy and limb lengthening |
|  |  | 14 | 2011 | Limb lengthening (tibia and femur) with Ilizarov apparatus |
|  |  | 14 | 2011 | Removal of external fixation device |
|  |  | 17 | 2014 | Removal of femoral implant |
|  |  | 17 | 2014 | Tibial corrective osteotomy with internal fixation |
|  |  | 20 | 2017 | Removal of implants |
| 07 | M | 6 | 2022 | Guided growth (distal femur, medial side) |
|  |  | 7 | 2023 | Implant removal and guided growth (distal femur and proximal tibia) |
|  |  | 8 | 2024 | Removal of implants |
| 08 | F | 4 | 2018 | Guided growth with 8-plate (proximal tibia) |
|  |  | 5 | 2019 | Removal of implants |
|  |  | 5 | 2019 | Removal of implants (repeat procedure) |

**Supplementary Table 4. Renal ultrasound findings of participants with X-linked hypophosphatemia (XLH)**

| **Patient No.** | **Nephrocalcinosis** | **Severity** | **Laterality** | **Age at detection (years)** |
| --- | --- | --- | --- | --- |
| 01 | Yes | Mild | Bilateral | 9.2 |
| 02 | Yes | Mild | Left | 5.0 |
| 03 | Yes | Mild | Bilateral | 17.6 |
| 04 | Yes | Mild | Not specified | 8.4 |
| 05 | Yes | Mild | Not specified | 8.3 |
| 06 | Yes | Mild–moderate | Bilateral | 7.4 |
| 07 | No | — | — | 10.1 |
| 08 | No | — | — | 11.5 |
